# Making the most of life: environmental choice during rearing enhances the ability of laying hens to take opportunities

**DOI:** 10.3389/fvets.2024.1425851

**Published:** 2024-06-14

**Authors:** Lena Skånberg, Regine V. Holt, Ruth C. Newberry, Inma Estevez, Kirste McCrea, Linda J. Keeling

**Affiliations:** ^1^Department of Applied Animal Science and Welfare, Swedish University of Agricultural Sciences, Uppsala, Sweden; ^2^Department of Animal and Aquacultural Sciences, Faculty of Biosciences, Norwegian University of Life Sciences, Ås, Norway; ^3^Neiker Basque Institute for Agricultural Research, Basque Research and Technology Alliance, Vitoria, Spain; ^4^IKERBASQUE Basque Foundation for Science, Bilbao, Spain

**Keywords:** pullets, behavioral development, environmental complexity, enrichment, litter, perches, positive animal welfare, agency

## Abstract

**Introduction:**

The potential of aviary housing for improving laying hen (*Gallus gallus domesticus*) welfare will be constrained if rearing conditions limit the hens’ behavioral ability to take opportunities. Incorporating theories on developmental plasticity and animal agency, this study aimed to determine: (1) whether a choice of litter and perch types during rearing would promote long-lasting changes in use of novel locations and resources, and (2) the influence of timing of choice provision.

**Methods:**

Laying hen chicks were assigned to either a “Single-choice” (one litter and perch type) or “Multi-choice” environment (four litter and perch types) during “Early” (day 1-week 4) and “Late” rearing (week 5–15). The environments were switched in half of the 16 pens in week 5, resulting in a 2 × 2 factorial design with four choice environment by period combinations. The allocation of perch and litter space was the same across all treatment combinations. In week 16, all groups were moved to standard aviary laying pens (Laying period, week 16–27).

**Results:**

When first moved to the laying pens, hens with Multi-choice in either or both rearing periods were quicker to spread out in their pen than hens with Single-choice throughout rearing. Multi-choice in Early rearing also reduced the latency to use novel elevated structures (perches and nests) in the laying pens. Multi-choice during Late rearing increased success in finding and consuming hidden mealworms (tested in weeks 9–17) and increased the proportion of eggs laid on elevated nesting trays. Numerically, hens switched from Multi-choice to Single-choice in week 5 used the outdoor range less than hens switched from Single-choice to Multi-choice.

**Discussion:**

These results support the hypothesis that offering multiple resource choices during rearing improves hens’ ability to make the most of new opportunities by being more proactive in exploring and exploiting newly available resources. In different opportunity challenges, hens showed positive outcomes in response to choice during Early, Late or both stages of rearing, suggesting that best results can be obtained by offering environmental choice throughout rearing.

## 1 Introduction

In nature, high motivation to explore and low fear of novelty during early life can be essential for learning to identify suitable food items and locate other essential resources (1–3). Exploration, which involves the approach toward sources of stimulation in the environment, is proposed to be motivated by the primary emotional system of “seeking” (4) and leads to experiencing rewards that promote associative learning (5). Exploration can be accompanied by neophilia, the preference for or attraction to novelty, which is suggested to be a trait independent from neophobia, the avoidance of unfamiliar stimuli (6). Greater neophilia, especially toward potential food items, can be a good indicator of success in a novel habitat (7, 8). However, seeking and exploiting novel resources comes with risks. For example, foraging in a location away from the rest of the group can increase predation risk (9), and there is a risk that a novel food may be poisonous. The cost–benefit trade-off between exploration and neophobia in wild populations is influenced not only by the riskiness of the environment (e.g., predator pressure), but also by the complexity of the habitat, with information-gathering probably being more important in more varied environments (2, 10, 11).

Higher exploration when young may promote somatic state adaptive developmental plasticity (12) and lead to a higher motivation to gather information throughout life, promoting both action-driven and competence-building agency (11). The resulting behavioral flexibility is thought to be adaptive by enabling the young individual to become better fitted to its adult environment (12, 13). While the risks of predation and consumption of poisonous food items are usually lower for domestic animals and captive wildlife than for wild animals, the development of agency could nevertheless also be important for them, especially if they are destined to be kept in complex adult housing systems or given outdoor access. Animals kept for human uses are often exposed to different sources of novelty across their lifetime, such as new feeds, routines, and housing environments. They need to be motivated to explore, attracted to novel resources, and behaviorally flexible to make the most of new opportunities (14, 15).

A more complex rearing environment for farm animals such as laying hens (*Gallus gallus domesticus*) could encourage the gathering of information and promote the development of spatial navigation skills (16–18) as well as spatial learning and problem solving in general (19–21). In other words, rearing in a complex environment could increase the likelihood that laying hens are sufficiently behaviorally flexible to make the most of the opportunities offered to them later in life, thereby contributing to long-term positive welfare. In experimental set-ups, complexity is often increased by adding resources and comparing the outcome with that from a more barren environment lacking these resources [e.g., (20–22)]. For laying hens, this can involve providing litter, perches, shelters, objects, and sensory stimuli (23). Such inputs during rearing can result in laying hens exhibiting greater exploration of novel objects (24) and greater range use (23). In nature, however, a more complex environment also involves greater variation within the same general resource type, such as having access to multiple food types, and multiple choices of substrates for foraging, perching, and nesting, rather than a single type of each. Providing laying hens with variation in relevant resources when young could increase the biological relevance of the environment, thereby stimulating opportunity-taking later in life beyond that typically observed in today’s production environments.

Different forms of resources such as litter and perches are preferred by chicks for different types of behavior already from their first week after hatch (25). Presenting resource variants in the home environment therefore has the potential to lead to greater activation of the seeking emotional system (26), promoting the formation of positive associations through choosing and using different resource variants, or microhabitats along environmental gradients, for different purposes (5). Indeed, when provided with four different litter and perch types during early rearing, chicks were better able to locate a novel food resource at 3 weeks of age (27), showed a higher level of locomotion in an unfamiliar environment, were better at solving a spatial task at 5 weeks of age (19) and also displayed a higher prevalence of positively-valenced behaviors (28), compared to chicks from a standard rearing environment providing only one litter type and one perch type. It appears that the promotion of “seeking” and experience of having choices can increase use of novel resources compared to that observed with the same resource quantity but without the variety.

The first weeks of life appear to be critical for the development of a well-functioning behavioral repertoire (e.g., foraging, dustbathing, perching) and may affect traits such as fearfulness and spatial ability in the long term (29–32). Nevertheless, young pullets destined for multi-tier systems are often enclosed in the aviary rows of the rearing facility during these first weeks of life, before being given full access to the complex aviary space including the litter floor and multiple tiers (24, 33). This practice ensures that they can easily find essential resources such as food and water that are only provided on aviary tiers. However, the lack of experience with other resources could suppress behavioral development during a sensitive window (34), with long-term repercussions for later use of novel resources such as food items, litter types, perch designs, nests, and outdoor range.

The aim of the present study was to investigate the long-term effects of rearing laying hens in an environment with greater variation, in this case an environment with a variety of litter and perch types. Based on the adaptive value of developmental plasticity (12) and agency (11), we hypothesized that access to an environment offering multiple choices during rearing would improve hens’ behavioral flexibility to make the most of new opportunities. Thus, we predicted that exploratory behavior and use of novel resources would be promoted by experiencing greater environmental choice during rearing. Further, based on a hypothesized early sensitive period, we predicted that hens having multiple environmental choices during the first 4 weeks of rearing would show responses to novel opportunities indicative of having greater exploratory motivation, greater neophilia, lower neophobia, and greater behavioral flexibility than hens only exposed to multiple environmental choices later during rearing. Given that responses to novel food items, novel objects, and novel environments are not always correlated (6, 7, 35), we evaluated laying hen responses to different sources of novelty and to opportunities that they are likely to encounter under commercial conditions at relevant ages.

## 2 Materials and methods

### 2.1 Ethics

This study was approved by the National Ethics Committee for Animal Experiments in Uppsala, Sweden (protocol code 5.8.18–11,549/2017, 28 July 2017).

### 2.2 Animals and housing

Day-old chicks (*n* = 364) of the white layer hybrid Bovans Robust were brought from a commercial hatchery to the rearing facilities at the Swedish Livestock Research Center in Uppsala. They were marked with individually numbered leg rings, weighed, and assigned to one of 16 pens (240 × 120 × 180 cm) in initial groups of 22 or 23 in a manner that ensured a similar average bird weight and uniformity in each pen. Leg rings were replaced by individually numbered wing tags (Jiffy bands, National Band and Tag Company, Newport, KY, United States) on day 17. Birds were kept in the same pen from day 1 until week 16, when each group was relocated to a laying pen in another building. Five chicks died or were euthanized for varied reasons prior to starting behavioral observations, leading to 23 birds in 8 pens, 22 birds in 7 pens and 21 birds in one pen throughout the study. Standard commercial feed (starter from day 1, grower from week 7, layer from week 16) and water were provided *ad libitum*. Temperature, lighting cycle and light intensity followed breeder recommendations [Hendrix Genetics (36)]. All days included a 15-min dawn and dusk period during the daily light: dark cycle. In the rearing pens, average light intensity was 18 lux at bird level (range: 7–37 lux), while in the laying pens, it was 5 lux at bird level (range: 3–8 lux). The walls of the pens in both the rearing and the laying pen were covered to visually block neighboring birds. Birds were checked daily during routine animal care.

During the first 4 weeks (Early rearing period), litter was presented in shallow plastic trays (71 × 35 × 3.5 cm) as well as on the concrete floor. A heat lamp was placed in the middle of the pen (centered over the trays). Perches (120-cm long) were initially 15 cm high and then raised at 3 weeks to 45 cm and in week 5 to 55 cm. From week 5 (Late rearing period), litter was provided only in plastic boxes (55 liters, 78 × 56 × 18 cm) in the same locations as the former plastic trays. In week 16, all groups were moved to a laying house (Laying period) and kept in 16 pens (362 × 356 × 297 cm) with a litter area (132 × 356 cm) and a raised (32 cm) plastic slatted area (230 × 356 cm). All laying pens had the same litter (crushed straw pellets), four mushroom-shaped plastic perches (heights 43, 96, 149 and 205 cm), a single wooden square-shaped perch (height 187 cm), three elevated open plastic nesting trays (71 × 35 × 3.5 cm, at heights 70, 155 and 220 cm), and two enclosed colony nests with a vinyl curtain door and plastic turf mat (115 × 46 × 30 cm) located on the slatted floor area. The experiment ended at 27 weeks when the birds were donated to hobby poultry keepers. The experiment was conducted between the 1^st^ of October 2019 and the 5^th^ of April 2020.

### 2.3 Environmental choice treatments

**Figure 1:**
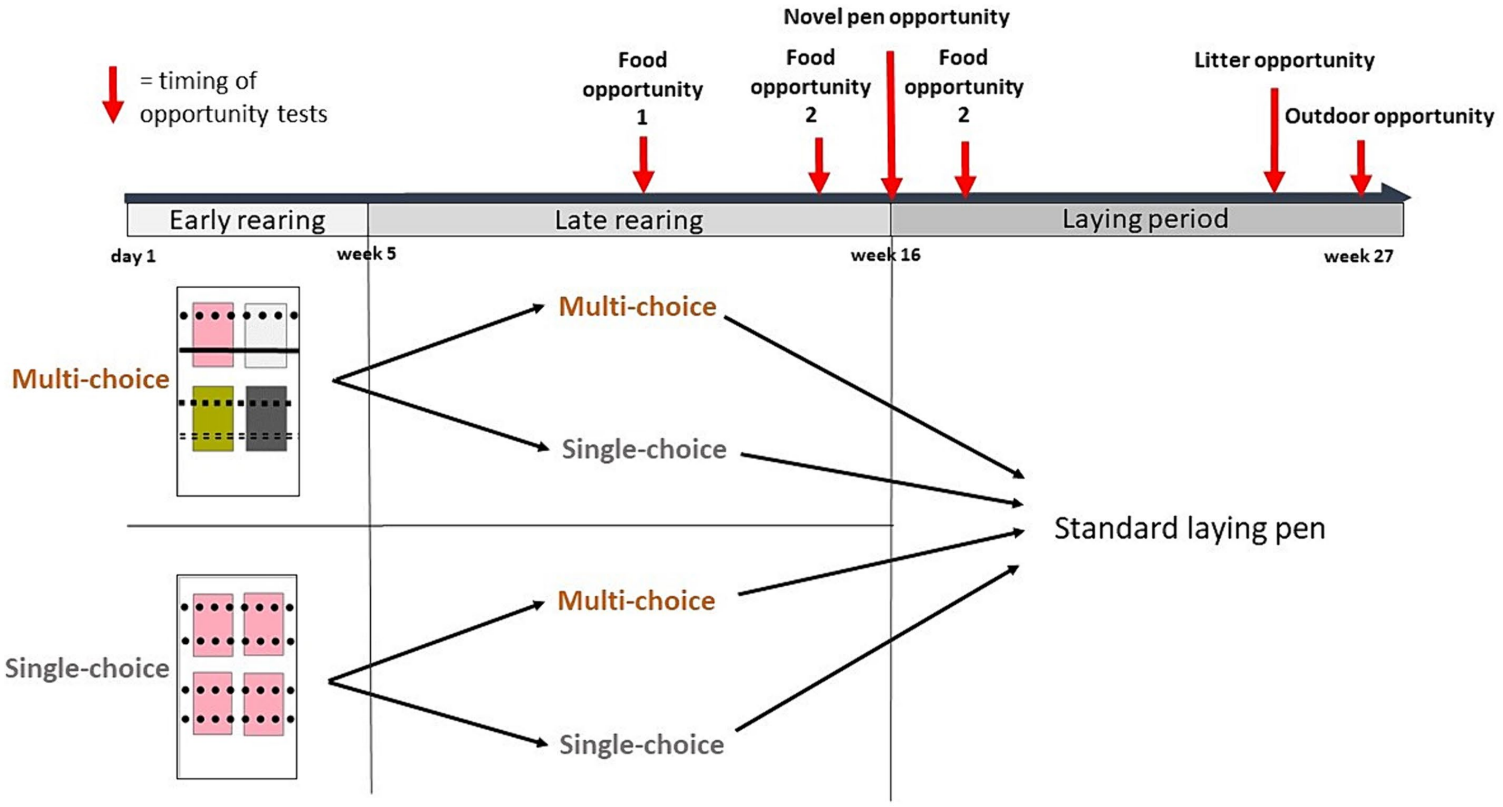
Two environments, Single-choice and Multi-choice, were provided during two rearing periods, Early (day 1 to week 4) and Late (week 5–15), resulting in four choice treatments during rearing. The rectangles illustrate a sample pen from each environment, with colored squares representing litter types and different line styles representing perch types. In week 16, the birds in each rearing pen were moved to a standard laying pen. Behavioral responses to different resource opportunities were investigated in different tests or contexts: food opportunities (in a test arena during weeks 9–10 and in the home pens in week 14 and 17, respectively), a novel pen opportunity (the 1 hour following relocation to the laying pens in week 16), a litter opportunity (in week 26), and an outdoor opportunity when birds were given outdoor access in the final week of the study (week 27). The timing of the different behavioral observations is illustrated with red arrows. Locations of laid eggs were also recorded throughout the laying period (weeks 16–27).

We compared two environments (Single-choice and Multi-choice) during two periods: Early (day 1-week 4) and Late (week 5–15) rearing. At all times, half of the rearing pens were Single-choice and the other half were Multi-choice. In week 5, half of the pens were swapped to the other environment, resulting in four choice by period treatment combinations: Single*Single, Single*Multi, Multi*Single, and Multi*Multi (Figure 1).

In the Multi-choice environment, there were four different types of litter and four different types of perches in each pen. The litter types were fine-grained sand (maximum 0.3 mm Ø), wood shavings (without dust), straw (wheat, long-cut) and peat (100% Sphagnum moss). The perch designs were round rubber with a wooden core (3.5 cm Ø), a flat wooden plank (9.5 × 2 cm), a swinging braided rope (4 × 3 cm) and flat wire mesh in a wooden frame (wood frame 2 × 1 cm, mesh 9.5 cm, mesh openings 1 cm^2^). The location of the different litter and perch types was balanced across pens. In the Single-choice environment, the litter type and perch type were the same at all four pen locations. We balanced potential effects of specific perch and litter types by providing all the types used in the Multi-choice environment across the Single-choice pens. We placed litter and perch types in each Single-choice pen so that, based on results from a pilot study, preferred litter types were paired with less preferred perch types and vice versa. This was done to avoid pens offering solely non-preferred or preferred litter and perch types. The pairings were: wood shavings-rope, sand-wooden plank, straw-rubber, and peat-wire mesh. The same environmental set-up was used in Nazar et al. (27).

### 2.4 Food opportunity

We conducted two sets of food opportunity tests involving locating and consuming an initially novel food reward. The first tests (food opportunity 1) took place under experimental conditions in an initially novel test arena, which is typical practice for evaluation of exploratory behavior [e.g., (37)]. We also conducted a second set of tests (food opportunity 2) in the home pens, following the recommendation of Réale et al. (38), who proposed that exploration of novel objects or food should be evaluated in familiar, ecologically relevant conditions, which for production animals would be their home environment. This method avoids the influence of fearfulness due to novelty of the test arena (39).

#### 2.4.1 Test arena (food opportunity 1)

**Figure 2:**
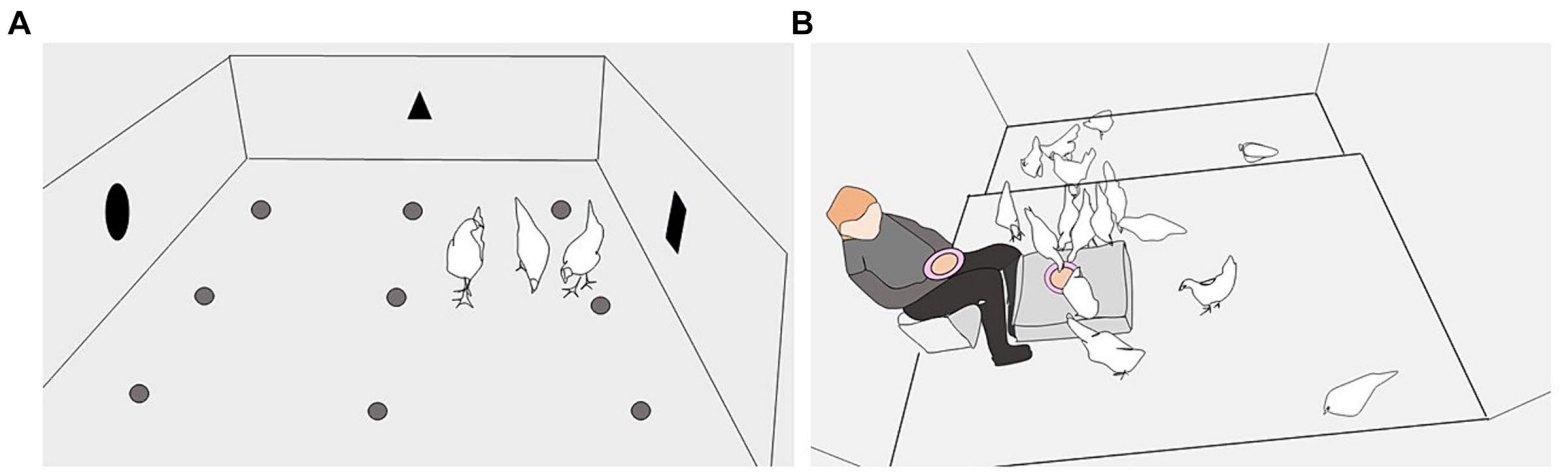
The two types of food opportunities. **(A)** Food opportunity 1 tests were conducted in an initially novel arena set up in the rearing house and containing nine plastic coffee cups on the floor, each baited with a live mealworm. Birds were tested individually or in trios and given 5 min to find and eat the mealworms. The illustration is of a trio session. **(B)** Food opportunity 2 tests were conducted in the home pen and birds were given 1.5 min to locate and consume mealworms hidden under crushed straw pellets in a “ground” bowl on the litter floor or on a box, and a bowl held in the lap of a familiar researcher. The illustration is of a test in the laying pen, with the “ground” bowl on a box.

The first set of food opportunity tests took place in weeks 9–10, after the birds had been kept approximately equal lengths of time in the Early and Late rearing environments, at an age by which red jungle fowl (*G. gallus*) forage independently of their mother (40). The tests were conducted over a 12-day period, with a maximum of two repetitions/day and four test days per bird. The arena was situated in the rearing house so that light intensity, temperature, and sounds were similar to the conditions in the rearing pens. The arena had a concrete floor (248 × 218 cm), wire netting roof and cardboard walls (70 cm height). Nine plastic coffee cups were glued to the floor, each baited with one live mealworm (*Tenebrio molitor* larva) (Figure 2A). The same three birds per pen were tested repeatedly. Three birds in each pen were selected in a standardized, semi-random way to encompass variation among birds within each pen. One bird was easy to catch, often directly approaching the researcher, one bird was lifted off a perch, and one bird was more difficult to catch due to more attempts to avoid the researcher. The three test subjects were marked with a specific leg ring color for ease of identification during the following test days.

The three test birds per pen were all tested under two social conditions: together (trios) in repetitions 1–4 and 6, and each bird individually in repetitions 5 and 7. For testing, the three subjects per pen were caught and taken together to the arena in a covered transport box. In individual sessions, each bird was placed alone in one corner of the test arena and given 5 min to find 9 mealworms. Any eaten mealworms were replaced before the second bird was placed alone in the test arena, and so on. In trio sessions, the three birds were placed together in the same corner of the test arena and given a maximum of 5 min to find 9 mealworms. A researcher sat outside the arena and registered when each mealworm was eaten and by which individual. If all mealworms were eaten before 5 min elapsed, the test was considered finished. After testing, the three birds were returned to their home pen in the transport box.

The number of mealworms eaten of the number provided was recorded for each repetition, and a pen score for the proportion of mealworms eaten was calculated. For example, if Bird 1 ate three mealworms, Bird 2 ate one mealworm and Bird 3 ate five mealworms, the pen score would be 0.33 (9 out of 27 possible) if tested individually, but 1.0 (9 out of 9 possible) if tested as a trio.

#### 2.4.2 Home pen (food opportunity 2)

The second set of food opportunity tests were conducted at group level, in the home pens, during week 14 (13 days before transition to the laying house) and week 17 (13 days after the transition). The test involved three repetitions of a 1.5-min opportunity for the birds in the pen to locate and consume mealworms hidden under crushed straw pellets in two baited bowls (pink porcelain, 22 cm Ø x 6 cm deep), each containing 10 mealworms. In the rearing pen, one bowl was placed on the ground and the other was in the lap of a researcher seated in the pen [see (27) for an illustration]. In the laying pen, we increased the challenge level by placing the “ground bowl” on a novel plastic box (78 × 56 × 18 cm; Figure 2B). The first two repetitions were conducted 3 h apart on the same day, while the third took place 24 h after the first repetition. Before each repetition in the rearing pen, the three birds tested previously in the arena were removed from the pen, as they had prior experience of mealworms. This meant that the crushed straw pellets, mealworms, and bowls were initially novel to all birds in the pen. In the laying period, all birds had previous experience with the mealworms and so all stayed in the pen during the test. The number of mealworms eaten of the 20 provided was recorded for each repetition, and the data were expressed as proportions.

### 2.5 Novel pen opportunity

**Figure 3:**
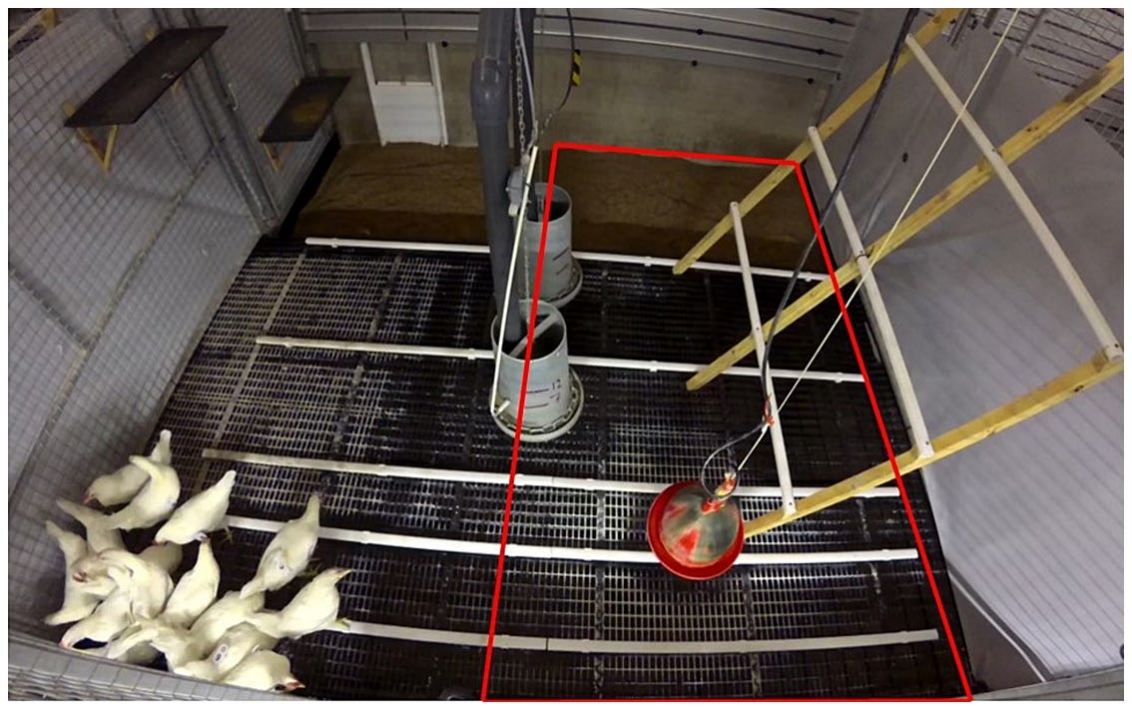
The novel pen opportunity. After birds from a rearing pen were released in one corner of their new laying pen, their behavior was recorded over their first hour in this novel environment. The pen contained a litter floor and (closed) pop hole at the back, a slatted floor at the front, two hanging feeders, a bell drinker, plastic and wooden perches, elevated nesting trays (two of three are visible on the left wall), and two colony nests at the front (not visible). Latencies of the first and fifth bird to use these novel resources, as well as counts of birds on the litter and slats within the red rectangular area, were recorded.

The birds were video recorded during the first hour after they were relocated from the rearing to the adult laying pens (Figure 3). The laying pens had a standard design (see section 2.2) and the feeders, drinker, slatted area, perches, and enclosed colony nests were all novel while the elevated nesting trays and litter were partially novel. The nesting trays had been used as litter trays during the Early rearing period but were now without litter and set in a raised wooden frame attached to the wall giving them a novel appearance. The litter type in the laying pen (crushed straw pellets) had been used in food opportunity test 2 for a total of 4.5 min of possible previous exposure.

When introduced to their new pen, the birds were placed as a group on the slats. For each group, the latencies for the first and fifth bird to be observed feeding, on the litter, and on an elevated structure (perch or nest) were determined from the video recordings and thereby two latencies per pen were used for the analysis. If no bird used these resources, a maximum latency of 60 min was assigned for both latencies, while if fewer than five birds used them, 60 min was assigned as the fifth bird’s latency. As only two pens had five birds using an elevated structure during the 60-min observation period, only the latency for the first bird was analyzed for this variable.

To assess group-level exploration of the novel pen, the number of birds on the litter and slats in the opposite half of the pen from the initial placement was recorded using instantaneous scan sampling at 2-min intervals during the first hour immediately after being introduced. For data analysis, the 60-min observation period was divided into three 20-min phases and average count per scan per phase was expressed as a proportion of the total number of birds in the pen.

### 2.6 Nest opportunity

Eggs were collected once daily, and their locations and weights (to the nearest 0.1 g) were registered for each group from the first egg until the end of the experiment (excluding cracked or broken eggs). As a measure of how the birds used the pen space for ovi-position during the laying period, we counted the numbers of eggs laid on the elevated nesting trays, in the colony nests, and on the floor (“floor eggs” located on the slats or litter). For analysis, we calculated the proportion of eggs laid in each location (elevated nests, colony nests, on the floor).

To evaluate changes in egg production and egg weight over time, the 72-day laying period (week 16–27) was divided into three 24-day periods. For each pen, the average number of eggs produced per bird per 24-day period, and the average egg weight per 24-day period, were calculated for analysis.

### 2.7 Litter opportunity

**Figure 4:**
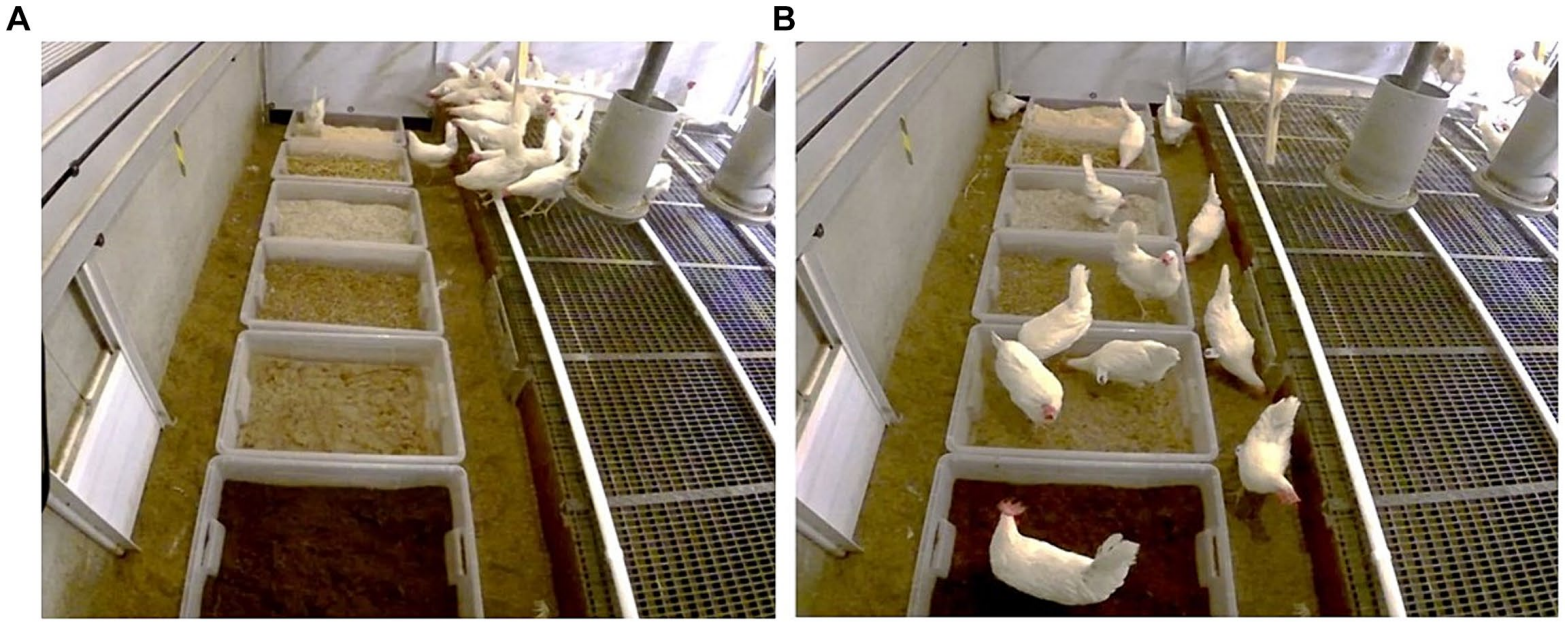
In the litter opportunity test, **(A)** the latency for the first hen to enter each box containing a different litter type was registered, and **(B)** the number of hens performing foraging, dustbathing or other behavior in each litter box was recorded at 1-min intervals for 1 h. Litter types were categorized as current, familiar from rearing, or novel.

This test was conducted in the adult home pen in week 26. Birds were given 1 h of access to six boxes (55 liters, 78 × 56 × 18 cm), each containing a different type of fresh litter (Figure 4): crushed straw pellets (current litter type in the laying pens), straw, peat, sand and wood shavings (the four litter types used during rearing) and hemp shavings (a novel litter type for all birds). Straw, peat, sand and wood shavings were all familiar to birds exposed to Multi-choice, whereas three types were novel to birds only exposed to Single-choice, with the novel types varying between groups. The location of the litter types within boxes was balanced across the pens. In each pen, the latency for the first bird to be seen in each litter box location was registered using continuous observation of a video recording of the test (Figure 4A). In addition, the number of birds performing foraging and dustbathing was registered by litter box location using instantaneous scan sampling of the video at one-minute intervals for 1 h (Figure 4B). For analysis of each behavior, the behavior count per box per scan was expressed as a proportion of the birds in the pen. The number of familiar and novel litter types varied across treatments. Therefore, for each latency and proportion variable three pen average values were calculated, one for each of the three degrees of litter familiarity (current, familiar from rearing, and novel, respectively) for use in the statistical analysis.

### 2.8 Outdoor opportunity

In week 27, a pop hole in each laying pen was opened giving each group access to a separate outdoor range (3.5 × 16 m) connected to the pen. This occurred in April during a period of sunny weather with no rain. Each range had wire-netting sides and roof, fine gravel with some short vegetation (mainly grass) and was divided into four equal areas (3.5 × 4 m) representing the nearest to farthest section from the house wall. On the first 3 days, the pop holes were open for a total of 12.5 h, during which instantaneous scan sampling was performed every 30 min by direct observation. The latency for the first bird per pen to be seen outdoors was registered to the nearest 30-min observation interval, expressed as a value from 0 to 12.5. The number of birds in each range section per scan was also recorded. However, few birds used the range over the 3 days of observation, so these data were combined and analyzed as the average number of birds outdoors per scan.

### 2.9 Statistical analysis

Analyses were conducted in R Studio (Version 1.3.959). A description of the statistical model for each variable is provided in Supplementary Table S1. The statistical models always included the Early rearing environment (Single-choice or Multi-choice), Late rearing environment (Single-choice or Multi-choice), and their interaction (Early Single*Late Single, Early Single*Late Multi, Early Multi*Late Single and Early Multi*Late Multi, to assess whether choice effects changed across rearing periods). For variables with repeated measurements, pen was included as a random effect. Additional fixed effect factors were included in models for specific variables. The social condition (Individual or Trio) was included in the model for food opportunity 1, and the test period (Rearing or Laying) for food opportunity 2. Order (first or fifth bird) was included when examining latencies to use resources (litter and feed) in the novel pen. The models evaluating egg production variables included 24-day period (1st, 2nd, or 3rd), while the model examining the proportion of eggs laid in specific locations also included egg location (Colony nests, Elevated nests, or Floor). The litter opportunity model included the degree of familiarity with the litter types (Current, Familiar from rearing, or Novel). Models with an additional factor also included the interaction of that factor with the choice environment, within and across rearing periods, but if these interactions had *p* ≥ 0.10, they were dropped from the final model.

All variables were regarded as continuous values if fulfilling assumptions for normality and homoscedasticity. Repeated measures were analyzed in linear mixed models with restricted maximum likelihood using the packages lme4 (41), pbkrtest (42) and lmerTest (43). Significance was assessed using Type III Wald F tests with the Kenward-Roger approximation of degrees of freedom. When there was only one value per pen, linear models were employed (lm-function), with significance assessed using Type III tests and the contr-sum function. Assumptions for fitted models were checked visually in probability plots of residuals (QQ plot) and plots of residuals versus fitted values. When assumptions were not met for a linear model, data calculated as averages per pen were analyzed using Kruskal Wallis tests. Few pens had dustbathing birds during the litter opportunity test and only descriptive data are presented for this variable.

Results are presented as estimated marginal means with standard error and plotted using ggplot2-package (44). Transformed values are presented after back-transformation to the original scale. The significance level was set at 0.05. Significant results are presented with planned pairwise comparisons made using the emmeans-package (45). For all variables, overall means with SE are presented for each treatment combination (Single-choice or Multi-choice in the Early and Late rearing periods, respectively; Supplementary Table S2).

## 3 Results

**Figure 5:**
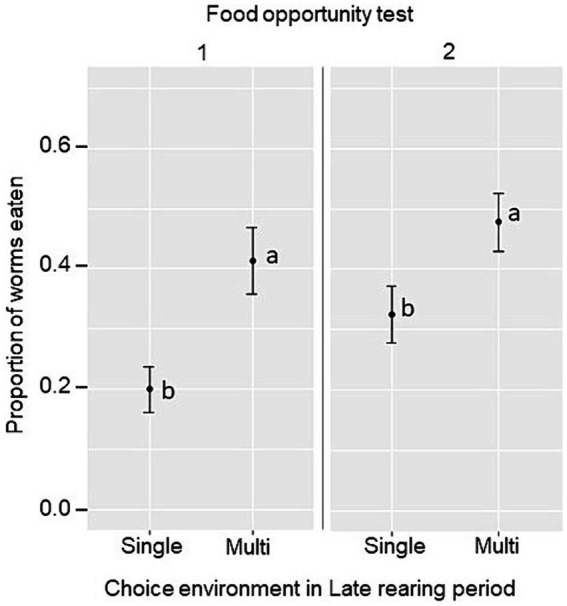
Estimated marginal mean ± SE proportion of mealworms eaten per group in the food opportunity test 1 (test arena) and 2 (home pen) by birds kept in a Single-choice or Multi-choice environment during the Late rearing period. Different letters (a, b) indicate significant differences within each food opportunity test (*p* < 0.05).

In the two sets of food opportunity tests, the proportion of mealworms eaten was used as an indicator of the birds’ ability to exploit an initially novel food item in the test arena and in the home pen, respectively. The Late rearing environment was found to have a main effect on mealworm consumption, whereby the consumption was higher in birds exposed to the Multi-choice environment during the Late rearing period in both sets of tests (food opportunity 1: Single-choice, 0.20 ± 0.04; Multi-choice, 0.41 ± 0.06; F_1,12_ = 10.4, *p* = 0.007; food opportunity 2: Single-choice, 0.33 ± 0.05; Multi-choice, 0.48 ± 0.05; F_1,12_ = 5.11, *p* = 0.043, Figure 5). In addition, a larger proportion of mealworms was consumed in food opportunity 1 when birds were tested in groups of three (0.40 ± 0.04) than individually (0.21 ± 0.03; F_1,12_ = 33.64, *p* < 0.001), and in food opportunity 2 after relocation to the laying pens (rearing pen, 0.30 ± 0.04; laying pen, 0.51 ± 0.04, F_1,12_ = 17.87, *p* < 0.001).

**Figure 6:**
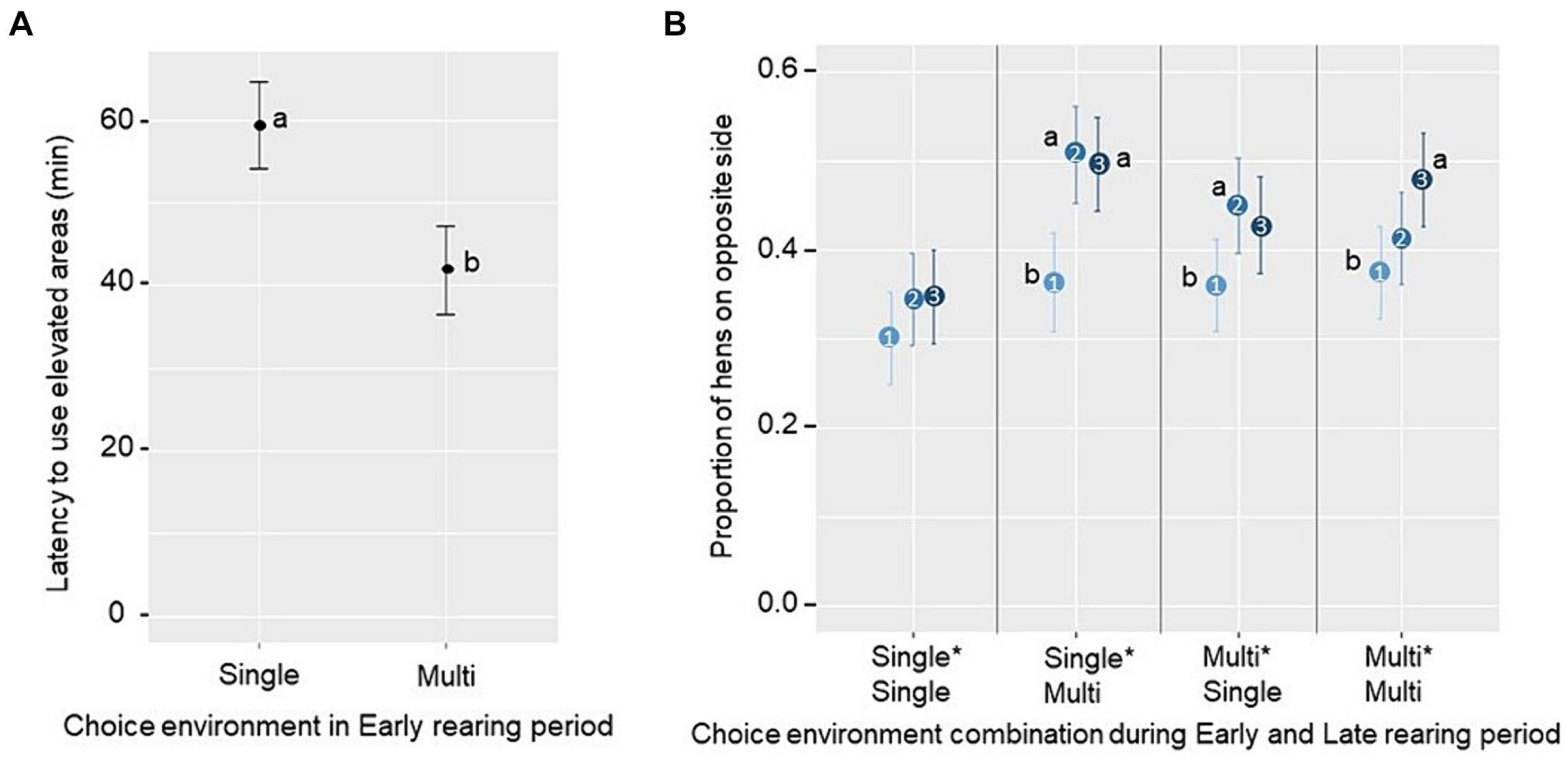
Estimated marginal mean ± SE of **(A)** latency for the first hen to use an elevated structure (perch or nest) in the first hour after relocation to the laying pen (novel pen opportunity) across groups reared in a Single-choice or a Multi-choice environment during Early rearing and **(B)** the proportion of hens being located at the side opposite to the entry during the first three 20-min phases (1, 2 and 3, ranging from light to dark blue, respectively) across groups from different choice environment combinations during Early and Late rearing period. Different letters (a, b) indicate a significant difference **(A)** between or **(B)** within a treatment (*p* < 0.05).

Latencies to use the novel resources in the laying pen were determined during the first hour after transfer from the rearing pen. No significant effects of the Early or Late rearing environment, or their interaction, were found regarding latencies for the first and fifth bird to feed or to be seen on the litter (*p* ≥ 0.10; see Supplementary Table S2 for treatment averages). However, there was a main effect of the Early rearing environment on latency for the first bird to use elevated structures, with the latency being shorter for groups reared in a Multi-choice environment (F_1,12_ = 5.49, *p* = 0.037; Figure 6A). Regarding the proportion of birds exploring the litter and slats located on the opposite side of the laying pen to the one in which they were introduced, there was a tendency for a three-way interaction between the Early rearing environment, the Late rearing environment, and the phase of observation (1st, 2nd and 3rd 20-min; F_2,425_ = 2.37, *p* = 0.094). Pairwise comparisons showed that, in groups from all treatment combinations except the Single*Single treatment, there was an increase in the proportion of birds on the opposite side of the pen from the first to the second phase (Single*Multi, *t* = 3.75 *p* < 0.001; Multi*Single, *t* = 2.49 *p* = 0.036), and/or the first to the third phase (Single*Multi, *t* = 3.5 *p* = 0.002; Multi*Multi, *t* = 3.11 *p* = 0.006; Figure 6B).

**Figure 7:**
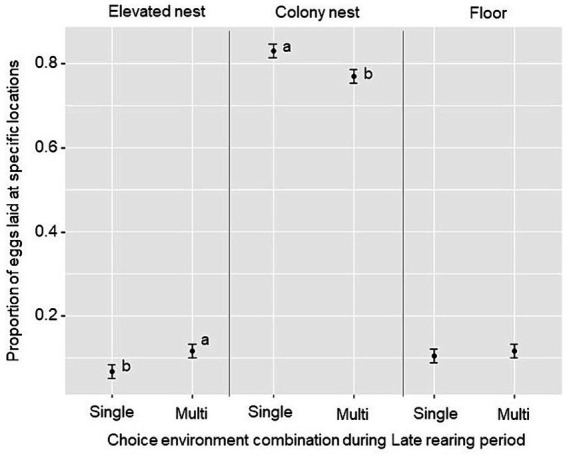
Nest site usage (estimated marginal mean ± SE) during the laying period (weeks 16–27) for the proportion of eggs laid in the elevated nesting trays, colony nests and on the floor by birds reared in the “Single-choice” and “Multi-choice” environments during the Late rearing period. Different letters (a, b) show a significant treatment difference (*p* < 0.05) within each egg location.

While most eggs were laid in the colony nests in all groups, a main effect of the Late rearing environment was found for nest use (F_2,114_ = 5.64, *p* = 0.005). Birds from the Multi-choice environment laid a lower proportion of eggs in the colony nests (*t* = −2.59, *p* = 0.012), and a higher proportion on the elevated nesting trays (*t* = 2.08, *p* = 0.041), compared to birds from the Single-choice environment during the Late rearing period (Figure 7). No treatment differences were found for the proportion of eggs laid on the floor, average number of eggs per bird or average egg weight per 24-day period (*p* ≥ 0.10, see Supplementary Table S2 for treatment averages).

**Figure 8:**
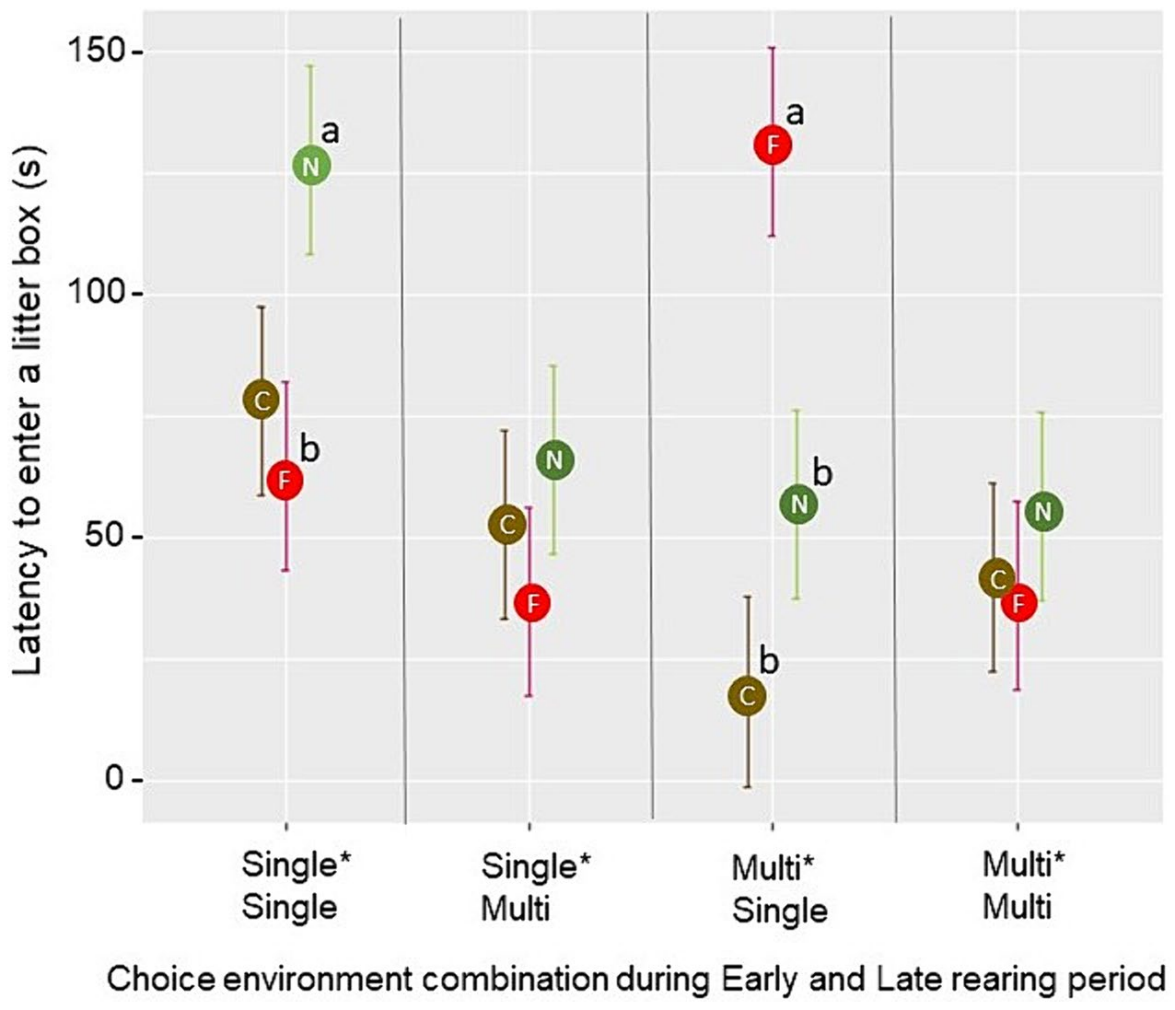
In the litter opportunity test, mean ± SE latency for the first bird to enter a test litter box was influenced by the degree of familiarity with the litter types in the boxes (C = current (brown), F = familiar from rearing (red), N = novel (green)) and rearing treatment. Different letters (a, b) indicate a significant difference (*p* < 0.05) within the choice environment combination during Early and Late rearing period across litter boxes of varying familiarity.

The latency for the first bird to enter a litter box during the litter opportunity test was affected by the three-way interaction between the Early rearing environment, the Late rearing environment, and the degree of familiarity with the litter type in the box (F_2,24_ = 5.25, *p* = 0.013). Pairwise comparisons revealed that, in pens from the Single*Single treatment, the latency for the first bird to explore a novel litter type was longer than the latency for the first bird to explore a litter type that was familiar from rearing (*p* = 0.018). In pens from the Multi*Single treatment, the latency for the first bird to enter a box containing a litter familiar from rearing was longer than the latency to enter a box containing a novel or the current litter type (*p* < 0.01; Figure 8). Latencies observed in the Single*Multi and Multi*Multi treatment groups did not increase for any of the three degrees of litter familiarity (*p* > 0.05). No significant effects of the choice environment in either rearing period were found for foraging in the litter boxes (*p* > 0.05). However, foraging was affected by the degree of familiarity with the litter (F_2,30_ = 7.91, *p* = 0.002), with a lower mean proportion of birds per scan foraging in a novel litter type (0.021 ± 0.004) than in the current litter type (0.040 ± 0.005; *t* = −3.06, *p* = 0.005) or a litter type familiar from rearing (0.045 ± 0.006; *t* = −3.73, *p* < 0.001). Dustbathing was seen in five pens (one Single*Single-pen with 5 observations, two Single*Multi-pens with 47 observations and two Multi*Multi-pens, with 84 observations in total). All of these dustbathing observations were seen in peat when peat was a litter type familiar from rearing.

The choice environment during the Early or Late rearing period did not affect the latency until the first bird per pen was seen on the outdoor range after opening the pop holes (*p* ≥ 0.10), or the average number of birds using the outdoor range per scan (*p* ≥ 0.10, see Supplementary Table S2 for treatment averages). Based on descriptive data, birds from all four Single*Multi pens visited the outdoor range at some point during the observations. Birds from only one of the Multi*Single treatment pens and from only two of the Multi*Multi and Single*Single treatment pens, respectively, were seen outside. Furthermore, in only two pens (both Single*Multi treatment pens) were the birds observed to move further away from the house than the first range section (i.e., beyond the nearest 25% of the total available range).

## 4 Discussion

The results of this study supported our first prediction that providing young laying hens with access to a variety of litter and perch types (i.e., offering multiple choices) during rearing, would have long-term effects on the birds’ abilities to capitalize on new opportunities presented to them. This was evident from the higher usage of resources such as novel food, and elevated nests, and faster exploration of the novel laying pen. In addition, the age of the birds when this variety was provided during rearing had an effect. Exposure to a choice of litter and perch types during the first 4 weeks increased use of novel three-dimensional pen space during the first hour after transfer to the laying pens. However, potentially beneficial effects of the choice environment on outcomes in the food and litter opportunity tests and elevated nest use were observed among birds that had been exposed to this variation during weeks 5–15, contrary to our prediction regarding the importance of environmental choice during the first 4 weeks after hatch. We discuss these results, but also how experience of different variants of the same resource may act in a more general way on bird behavior.

Irrespective of characteristics of the later rearing environment, environmental choices during the first 4 weeks of age (Early Multi-choice), increased birds´ readiness to perch following relocation to the novel laying pen (novel pen opportunity) as shown by the reduced latency to use elevated structures. There are several possible explanations for this finding. The perch types during rearing were visually different, which could have improved the birds´ ability to detect and identify an elevated structure in the novel pen as a possible perching location (46). The early experience with different perch types, and consequent improved balance (25), may have facilitated movement in three-dimensional space to a greater extent than providing just one type of perch during rearing (30, 47). The reduced latency to use elevated structures may have also been influenced by enhanced bone and muscle development, which has been reported in birds reared with perches (48, 49), and may have been further enhanced by balancing on different types of perches in the Multi-choice environment. Improved spatial navigation skills may have been associated with the sensitive period for brain synapse formation in the 3 weeks after hatch (50, 51). Overall, our current results can be interpreted as support for the existence of a short window early in life for birds to acquire foundational perching skills, and we add that acquisition of these perching skills seem to be further enhanced by the provision of different perch types during this window.

Gunnarsson et al. (52) reported that early perch access was connected to later nest use, as hens without perches within the first 4 weeks of life laid a higher percentage of eggs on the floor rather than in nests. In the present study, there were no treatment differences in the percentage of floor eggs, perhaps because all birds had early access to perches. But we did find that birds from the Late Multi-choice pens (reared with four litter and perch types in weeks 5–15), laid more eggs on the elevated nesting trays and fewer in the colony nests compared to birds reared with only a single litter and perch type during the same rearing period (Late Single-choice). This finding suggests that, even in the absence of early choice, later rearing experience in using varied elevated structures can impact the way in which birds exploit three-dimensional space as adults. Nest selection is probably determined by a combination of preferences for height (53), nesting material (54) and nest familiarity (55), among other factors. In feral hens, increased hatchability and initial offspring survival has been associated with elevated nesting sites (56). Nest use would need to be investigated further to assess the possibility that Late Multi-choice birds are more likely to use elevated nests than Late Single-choice birds at times when colony nests are occupied, thereby reducing competition for the most preferred nest space (57).

Birds reared with access to a variety of litter materials and perches between 5 and 15 weeks (Late Multi-choice) consumed more mealworms compared to birds reared with only one litter and perch type during this period (Late Single-choice). Increased success in locating and consuming mealworms was found in all food opportunity tests, irrespective of whether they were conducted during the Late rearing period or during the laying period. We did not test the effect of access to a variety of litter and perches on success in consuming mealworms during the Early rearing period since this was already shown by Nazar et al. (27). The birds from the same treatment (Late Multi-choice) showed a consistent, short latency to enter different litter boxes containing fresh litter of varying degrees of familiarity when briefly given access. In contrast, birds from the Late Single-choice pens displayed increased latencies. In combination, these results support the view that a Multi-choice environment stimulates the motivation to explore, thereby facilitating information gathering (3, 7) and positive affective engagement (58). These outcomes presumably promoted a sense of agency (11), improving the birds’ potential to find and make use of a hidden food resource as well as nest sites and a foraging and dustbathing (litter) resource. A spatially complex environment has been demonstrated to improve learning and working memory in spatial tasks (20, 59, 60), potentially by greater neural activation in the related brain regions. Even if the brain synapses are already formed, brain maturation continues until the 10th week after hatch (i.e., well into our Late Multi-choice period) when synaptic circuits are fine-tuned and most sensitive to environmental inputs (50), supporting the large effects we see from the Late rearing environment.

As effects of the Early rearing environment did not override the effects of the Late rearing environment for the majority of our measured variables, we suggest that the experience of losing choices through a reduction in the variety of perch and litter types in week 5 (Early Multi-choice and Late Single-choice) suppressed birds’ motivation and/or ability to take opportunities, while an increase in the number of choices in week 5 (Early Single-choice and Late Multi-choice) enhanced birds’ motivation and/or ability to take opportunities. The contrast effects found in our study are in line with affective trajectories, whereby individuals’ affective states are influenced by their expectations of the environment and a loss of resource or commodity can lead to negative affective states and vice versa (61). Although Paul et al. (62) did not find evidence to support a change in overall affective states in adult laying hens that gained or lost a richer environment, it is possible that a reduction or an increase in environmental complexity at week 5 has greater consequences for the highly active young pullet than a change later in life. Given that imprinting occurs during the first days after hatch and chicks stay close to their mother during at least the first 4 weeks after hatch (63, 64), the chicks may have developed an attachment to features of the Multi-choice environment during the Early rearing period. Furthermore, based on research in humans, Atkinson et al. (50) suggest that disturbances during the brain maturation phase (which in laying hens occurs between weeks 3 and 10), can lead to neurodevelopmental disorders. It is possible that the experienced loss of the Multi-choice environment when switched to the Single-choice environment in week 5 had a negative influence of brain development, with consequences related to their abilities and motivation to engage with novel opportunities.

The only result clearly influenced by the Multi-choice environment irrespective of when it was provided during rearing (i.e., during the Early period, Late period, or both) was the speed at which birds spread out over the floor when first placed in the laying pen. Birds with any previous experience of variation in the litter or perches were quicker to explore the whole area of the novel laying pen than birds experiencing only a single type of litter and perch. Birds reared in the Single-choice environment throughout rearing (Early and Late Single-choice) had the worst outcomes. The level of exploration shown by an individual (measured as locomotion in a novel arena) as a chick is linked to the level shown as an adult in the Red jungle fowl (37). Thus, stimulating exploration by giving birds experience of different resource variants at some point during rearing is likely to have effects over an even longer time than was studied in our experiment.

In this study we took the novel approach of focusing on the extent to which birds took opportunities when offered, which is different to what has commonly been done when investigating exploration or behavioral flexibility [e.g., (6, 7, 35)]. The reason for this was to explore the potential of utilizing a bird’s agency as a way to improve its own welfare (11, 65) and perhaps promote positive animal welfare. We propose that the differences in how birds used the opportunities given in this study, including quickly finding a safe place to rest (an elevated area), exploring the space and finding new sources of food, reflected differences in their motivation for these specific activities, as well as their general ability to exploit opportunity through increased cognitive abilities and motor skills. The originality in this study is the proposal that without motivation or the ability to take an opportunity, reducing fearfulness and neophobia alone will not increase the use of novel resources. A bird lacking skills to move in three-dimensional space, for example, is less able to use a safe perch even if motivated to do so and a bird with low fearfulness and neophobia will not explore a new outdoor range unless motivated to do so.

In conclusion, a rearing environment providing choices of relevant resource variants can potentially lead to positive animal welfare by promoting animal agency and positive affective engagement. We propose that such experience increases the motivation and ability of birds to exploit novel situations, building agency that makes it more likely that they will be able to make the most of opportunities when moved to a different environment or encountering new resources. The results of our study suggest that it is important for young birds to experience choices of certain biologically relevant resources such as perch types during the first 4 weeks of life. Providing choices within resource types later during rearing also shows benefits. Thus, providing a multi-choice environment during the whole rearing period can cash in on all the positive developmental plasticity effects observed in this study.

## Data availability statement

The datasets generated and R codes used for this study can be found in the Mendeley data respository, doi: 10.17632/wczdpy85k3.1.

## Ethics statement

The animal study was approved by National Ethics Committee for Animal Experiments in Uppsala, Sweden. The study was conducted in accordance with the local legislation and institutional requirements.

## Author contributions

LS: Writing – review & editing, Writing – original draft, Visualization, Supervision, Methodology, Investigation, Formal analysis, Conceptualization. RH: Methodology, Writing – review & editing, Investigation, Conceptualization. RN: Writing – review & editing, Supervision, Resources, Funding acquisition, Conceptualization. IE: Writing – review & editing, Funding acquisition, Conceptualization. KM: Writing – review & editing, Methodology, Investigation. LK: Writing – review & editing, Supervision, Resources, Project administration, Methodology, Investigation, Funding acquisition, Conceptualization.
