# Supplementary material for: Making the most of life: environmental choice during rearing enhances the ability of laying hens to take opportunities
Source: Front Vet Sci. 2024 Jun 14;11:1425851. doi: 10.3389/fvets.2024.1425851 (PMC11211632; doi:10.3389/fvets.2024.1425851)
Supplement: Supplementary file 1 [file Table_1.DOCX]

| Opportunity test | Output variable | Measure | Final models | Model |
| --- | --- | --- | --- | --- |
| Food opportunity 1 | Proportion of eaten mealworms | Average per pen per social condition (Individual/Trio) | Early + Late + Early*Late +  Social condition (Individual/Trio) + Pen ID (random effect) | LMM (sqrt) |
| Food opportunity 2 | Proportion of eaten mealworms | Proportion per pen per repetition | Early + Late + Early*Late +  Test period (Rearing/Laying) + Pen ID (random effect) | LMM |
| Novel pen opportunity | Latency to feed | Latencies for 1^st^ and 5^th^ bird to be seen pecking in feed in each pen | Early + Late + Early*Late +  Bird order (1st/5th) + Pen ID (random effect) | LMM (log) |
|  | Latency to be in litter | Latencies for 1^st^ and 5^th^ bird to be seen in the litter in each pen | Early + Late + Early*Late +  Bird order (1st/5th) + Pen ID (random effect) | LMM (log) |
|  | Latency for a bird to be on an elevated structure | Latency for the 1^st^ bird to be seen on an elevated structure in each pen | Early + Late + Early*Late | LM |
|  | Proportion of birds on opposite site of pen to initial placement per scan | Proportion per pen each 2-minute scan | Early + Late + Early*Late + Early*Phase (1^st^-3^rd^ 20 minutes)+Late*Phase + Early*Late*Phase+ Pen ID (random effect) | LMM |
| Nest opportunity | Proportion of eggs in specific locations | The average proportion of eggs laid in colony nests/elevated nests/floor (litter and slats) per pen per 24-day period (1^st^/2^nd^/3^rd^) | Early + Late + Early*Late + Egg location (colony/elevated/floor) + 24-day period (1^st^/2^nd^/3^rd^)  + Egg location*Early + Egg location*Late + Egg location*Early*Late + Egg location*24-day period+ Pen ID (random effect) | LMM |
|  | Number of eggs laid per bird | Average number of eggs per hen in each pen and day | Early + Late + Early*Late + 24-day period (1^st^/2^nd^/3^rd^) + Pen ID (random effect) | LMM |
|  | Weight per egg | Average egg weight in each pen and day | Early + Late + Early*Late +24-day period (1^st^/2^nd^/3^rd^)+ 24-day period*Early + 24-day period*Late + 24-day period*Early*Late +  + Pen ID (random effect) | LMM |
| Litter opportunity test | Latency to enter a test litter box | Average latency for the first bird to be seen in a test litter box of each degree of familiarity (Current/Familiar from rearing/Novel)in each pen | Early + Late + Early*Late + Early*Familiarity (Current/Familiar from rearing/Novel)+Late* Familiarity + Early*Late*Familiarity+ Pen ID (random effect) | LMM |
|  | Proportion of birds foraging in a test litter box per scan | Average proportion of birds foraging in a test litter box of each degree of familiarity (Current/Familiar from rearing/Novel) in each pen per 1-minute-scan | Early + Late + Early*Late + Early + Familiarity (Current/Familiar from rearing/Novel) + Pen ID (random effect) | LMM (sqrt) |
| Outdoor opportunity | Latency to observe first bird on the outdoor range | Latency for first bird to be seen outside in each pen (nearest 30-minute interval, range 0-12.5) | Effects of Early, Late and Early*Late separately in three tests | Kruskal Wallis |
|  | Number of birds on the outdoor range | Average per pen per scan | Early + Late + Early*Late | LM (log) |

Supplementary Material

# Supplementary Table

*Supplementary Table 1. Overview of the different statistical models. Model abbreviations: LMM= Linear mixed model, LM=Linear model, including specification of response variable transformations where needed: sqrt=square root, log=log transformation. Further descriptions of these models are found in the manuscript.*
