# Supplementary material for: Making the most of life: environmental choice during rearing enhances the ability of laying hens to take opportunities
Source: Front Vet Sci. 2024 Jun 14;11:1425851. doi: 10.3389/fvets.2024.1425851 (PMC11211632; doi:10.3389/fvets.2024.1425851)
Supplement: Supplementary file 2 [file Table_2.DOCX]

Supplementary Material

# Supplementary Table

Supplementary Table 2. Estimated marginal mean±SE for each variable by four rearing treatment combinations (Single-choice or Multi-choice in the Early and Late rearing periods, respectively). Presented values are back-transformed if response variables were transformed in the model.

| Measurement | Single*Single | Single*Multi | Multi*Single | Multi*Multi |
| --- | --- | --- | --- | --- |
| *Food opportunity 1 & 2* | | | | |
| Proportion of mealworms eaten (1; Test arena) | 0.24±0.06 | 0.39±0.08 | 0.16±0.05 | 0.44±0.08 |
| Proportion of mealworms eaten (2; Home pen) | 0.33±0.07 | 0.47±0.07 | 0.32±0.07 | 0.49±0.07 |
| *Novel pen opportunity* | | | | |
| Latency (s) for the first 5 birds (average of latency for the 1^st^ and 5^th^ bird) to feed | 2.65±0.71 | 2.79±0.75 | 2.98±0.80 | 2.98±0.80 |
| Latency (s) for the first 5 birds (average of latency for the 1^st^ and 5^th^ bird) to be seen on litter | 18.44±9.52 | 9.56±4.94 | 21.79±11.25 | 15.47±7.99 |
| Latency (min) for first bird to use an elevated structure | 60.0±7.49 | 58.9±7.49 | 38.8±7.49 | 45.0±7.49 |
| Proportion of birds observed on opposite side of pen to initial placement | 0.33±0.05 | 0.46±0.05 | 0.41±0.05 | 0.42±0.05 |
| *Nest opportunity* | | | | |
| Proportion of eggs laid on elevated nesting trays | 0.10±0.02 | 0.10±0.02 | 0.04±0.02 | 0.13±0.02 |
| Proportion of eggs laid in colony nests | 0.82±0.02 | 0.79±0.02 | 0.84±0.02 | 0.75±0.02 |
| Proportion of eggs laid on floor (litter and slats) | 0.08±0.02 | 0.11±0.02 | 0.12±0.02 | 0.12±0.02 |
| Number of eggs laid per bird (per 24-day period) | 15.7±0.54 | 16.1±0.54 | 16.2±0.54 | 15.6±0.54 |
| Weight/egg (g) | 47.1±0.44 | 47.5±0.44 | 47.5±0.44 | 46.6±0.44 |
| *Litter opportunity test* | | | | |
| Latency to enter a test litter box (s) | 89.4±14.8 | 51.9±14.8 | 68.8±14.8 | 45.4±14.8 |
| Proportion of birds foraging in a test litter box/scan | 0.03±0.01 | 0.04±0.01 | 0.03±0.01 | 0.03±0.01 |
| *Outdoor opportunity* | | | | |
| First 30-minute interval when first bird seen outside | 11.1±0.8 | 9.0±1.4 | 11.9±0.63 | 11.0±1.19 |
| Number of birds outside/scan | 0.005±0.01 | 0.37±0.71 | 0.001±0.002 | 0.004±0.01 |
